# Outcomes from the English National Lynch Syndrome transformation project

**DOI:** 10.1002/ijc.70330

**Published:** 2026-01-11

**Authors:** Kevin J. Monahan, Paul Fleming, Neil A. J. Ryan, Laura Monje‐Garcia, Ruth Armstrong, David N. Church, Jackie Cook, Fiona Lalloo, Sally Lane, Frank D. McDermott, Tracie Miles, Corinne Mallinson, Steven A. Hardy, Simone Gelinas, Francesca Faravelli, Frances Elmslie, Adam C. Shaw

**Affiliations:** ^1^ St Mark's Hospital Centre for Familial Intestinal Cancer London UK; ^2^ North Thames Genomic Medicine Service Alliance London UK; ^3^ Imperial College London London UK; ^4^ South East Genomic Medicine Service Alliance London UK; ^5^ Department of Gynaecology Oncology, Royal Infirmary of Edinburgh Edinburgh UK; ^6^ Centre for Reproductive Health, Institute of Regeneration and Repair, University of Edinburgh Edinburgh UK; ^7^ South West Genomic Medicine Service Alliance Exeter UK; ^8^ North West Genomic Medicine Service Alliance Manchester UK; ^9^ East of England Genomic Medicine Service Alliance Cambridge UK; ^10^ Wellcome Centre for Human Genetics, University of Oxford Oxford UK; ^11^ Central and South Genomic Medicine Service Alliance Oxford UK; ^12^ North East and Yorkshire Genomic Medicine Service Alliance Sheffield UK; ^13^ National Disease Registration Service (NDRS), NHS England Newcastle UK; ^14^ Guy's & St Thomas' NHS Foundation Trust London UK

**Keywords:** Colorectal Cancer, Endometrial Cancer, Genetic testing, Lynch syndrome

## Abstract

The English National Health Service (NHS) Lynch Syndrome Transformation Project was established to deliver universal testing for Lynch syndrome (LS) of newly diagnosed colorectal (CRC) and endometrial cancer (EC). A central aim was to integrate ‘mainstreamed’ genetic testing into routine care by cancer multidisciplinary teams in England. In June 2021 national and regional LS project teams were established to support ‘LS champions’ within local cancer multidisciplinary teams (MDTs). A comprehensive retrospective genomic dataset and dashboard was compiled by the National Disease Registration Service (NDRS), complimented with prospective local MDT audit (2022–2023). A robust national registry of people diagnosed with LS was developed to ascertain individuals for targeted interventions, including for a new national LS Bowel Cancer Screening Programme (LS‐BCSP). In total 276 LS champions were appointed and trained across 248 CRC and EC MDTs (>95% coverage nationally). Tumour Mismatch repair (MMR) tumour testing rates increased for CRC (43 >94%) and EC (19 >94%). MDT‐led mainstreaming services were developed in 46% of all CRC and 41% EC teams in England. In subgroup data, the time to germline genetic testing was 21 days in ‘mainstreamed’ patients, versus 180 days referred to regional clinical genetics services. New diagnoses of LS have consistently increased each year from a total of 545 in 2020 to a total of 1394 in 2024 (an increase of 255% vs. a target of >50%). The NHS England LS Transformation project has driven equitable nationwide delivery of diagnosis testing for Lynch syndrome, with integration of ‘mainstreamed’ genetic testing into routine cancer care.

## Graphical abstract

What's new?

The elevated cancer risk conferred by Lynch syndrome can be mitigated through preventive interventions. However, Lynch syndrome often goes underdiagnosed. The National Lynch Syndrome Transformation Project in England aims to ensure that patients newly diagnosed with colorectal or endometrial cancer are offered testing for the syndrome. After working with cancer multidisciplinary teams across the country, the authors report a 255% improvement in Lynch syndrome diagnosis between 2020 and 2024, identifying over 10,000 patients for a national registry. The project has driven equitable nationwide delivery of diagnosis testing for Lynch syndrome, with integration of mainstreamed genetic testing into routine cancer care.

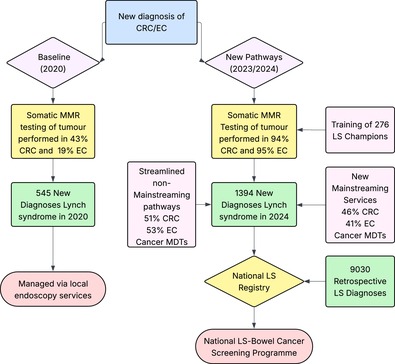

**Abbreviations**
BCSP: bowel cancer screening programme
CRC: colorectal cancer
EC: endometrial cancer
ENLSR: English National LS Registry
LS: Lynch syndrome
MDM: multidisciplinary teams meeting
MDT: multidisciplinary team
MMR: Mismatch repair
NHS: National Health Service
NICE: National Institute for Health and Care Excellence

## 1 INTRODUCTION

Lynch syndrome (LS) is an autosomal dominant inherited condition caused by pathogenic variants in DNA mismatch repair (MMR) genes *MLH1*, *MSH2*, *MSH6* and *PMS2*, or by deletions in *EPCAM* which modify the expression of MSH2.^1^ LS causes increased susceptibility to multiple cancers including colorectal (CRC) and endometrial (EC). However, this elevated cancer risk can be mitigated through a range of preventive interventions that significantly improve life expectancy. For individuals with LS who develop cancer, diagnostic molecular testing provides personalised treatment opportunities that further enhance survival.^2^, ^3^, ^4^, ^5^, ^6^, ^7^, ^8^

LS affects approximately one in 400 individuals^9^, ^10^ and therefore affects approximately 200,000 people in England, however, a national registry indicates that we have diagnosed fewer than 15,000 people with LS.^11^, ^12^, ^13^ As a result, many opportunities for cancer prevention and improved outcomes after diagnosis may be lost. A major contributor to this under‐diagnosis is the absence of clearly defined clinical responsibility, described in the literature as a ‘diffusion of responsibility.^14^, ^15^

In England, the National Institute for Health and Care Excellence (NICE) has recommended universal LS testing for all new colorectal cancer (CRC) cases since 2017 (DG27)^16^ and for endometrial cancer (EC) cases since 2020 (DG42).^17^ These guidelines specify tumour‐based triage—using either immunohistochemistry for mismatch repair (MMR) proteins or molecular testing for microsatellite instability—to identify patients who should be offered constitutional (germline) genetic testing. In parallel to publication of DG27, a UK national consensus^14^ recommended the development of a national LS registry, a quality assured colonoscopic surveillance programme, and a dedicated clinical champion for LS embedded within cancer multidisciplinary teams (MDTs), recommendations which have been developed into three inter‐related national projects in England.

The National Lynch Syndrome Transformation Project was established to support the implementation of universal tumour testing for LS in individuals diagnosed with CRC and EC, in line with NICE guidelines.^18^ The project focused on enabling secondary care teams to build robust, guideline‐compliant testing pathways. Central to its success was a comprehensive, multiprofessional education and upskilling programme, tailored to the needs of diverse cancer teams and regions. This was supported by regional and national transformation teams who helped identify barriers and co‐develop practical, context‐specific solutions. The project was underpinned by a national dataset developed in partnership with the National Disease Registration Service (NDRS), alongside local audit programmes that monitored testing rates, informed system improvements, and identified gaps in reflex tumour screening and referral for germline testing—whether through clinical genetics services or mainstreamed models within cancer MDTs.

## 2 METHODS

The national LS transformation programme methods have previously been reported.^18^ In summary, a national team led by two clinical leads, a national LS nurse and two project managers was formed. The national team arranged monthly national oversight group meetings which includes representation from 21 national cancer alliances and seven Genomic Medical Service Alliances (GMSAs), in addition to representatives from cancer programme and genomics unit teams at NHS England (NHSE). Each of seven GMSA regions appointed a clinical lead and a LS nurse to oversee training within each geography. The oversight group also included membership from specialist CRC and EC experts, NDRS, and patient representatives were appointed from the charities Lynch Syndrome UK, Bowel Cancer UK and the Eve Appeal.

The goals of the project included: A 50% increase on testing across each step of the testing pathway (MMR tumour testing, methylation or *BRAF* testing, and constitutional genetic testing) compared with baseline level measured by NDRS (Table S1). The baseline NDRS data represents comprehensive and robust mandatory pathology reporting patient level data as described in previous publications,^11^, ^12^ and from this a national reporting dashboard linked to individual cancer teams was developed.

The regional cancer alliances were asked to identify LS champions within each CRC and Gynae‐oncology (managing endometrial cancer [EC]) MDT. Each LS champion was responsible for coordination of specific roles within their team, ensure there were systems in place to identify and test eligible patents, and that these patients were offered appropriate testing. Regional teams developed tailored, quality improvement plans for each MDT, and multi‐level training programmes for the national workforce. A national training programme was developed to equip LS champions, cancer MDT clinicians, and regional support networks. This combined complementary online training modules supplemented by in‐person workshops delivered by regional LS nurses and GMSA teams. These in‐person sessions focused on co‐developing practical and relevant solutions to address team‐specific barriers and embed sustainable testing pathways.

Initially funded by NHS England for 1 year (2021/2022), early progress and demonstrable impact led to successive extensions of the project's funding into 2022/2023 and 2023/2024, with transitional support allocated for 2024/2025 to support the move towards integration into ‘business as usual’. The project extensions aimed to build on early progress and harness the strong engagement of partner GMSAs, Cancer Alliances, and local MDTs to accelerate the delivery of mainstreamed genetic testing across MDTs in England. These efforts drove improvements across the entire testing pathway—from initial tumour testing, through further somatic analysis, to germline genetic testing—surpassing the project's target of a 50% increase in testing rates at each step.

### 2.1 Delivery of the training programme

A multifaceted training programme was developed to support multi‐professional workforce development, using an evidence‐based pedagogical approach. The training focused on the identification of eligible patients, strategies to overcome barriers to testing, and the implementation of mainstreamed constitutional genetic testing within cancer services. National leads developed tailored online training modules for CRC and EC MDTs, primary care teams, and pathologists.^19^ These were complemented by national and regional workshops, providing further support to cancer MDTs and pathology teams.

Training delivery was achieved through a combination of central resources and local initiatives tailored to the requirements of local teams. In addition, the National team delivered online workshops/webinars through Microsoft Teams. Invites were distributed widely through Cancer Alliances and local networks.

Following module completion, regional teams engaged directly with individual cancer MDTs to deliver in‐person, one‐to‐one interactive workshops with clinicians, supporting practical implementation. As part of the training initiative, cancer teams were asked to audit 30 consecutive CRC or EC cases to identify local gaps or weaknesses in their testing pathway. The training modules were structured around two core topics: Module 1 focused on the identification of eligible patients and overcoming barriers to testing, while Module 2 addressed the delivery of mainstreamed constitutional genetic testing. Additional bespoke content was also developed for primary care professionals and for pathologists, in collaboration with the Royal College of Pathologists.

Local training needs were assessed through dialogue between MDT Lynch champions, Cancer Alliance programme managers, the Regional team leads (medical and nursing), and local Clinical Genetics services. Such integration was deemed essential given the recognition that genetic testing for Lynch syndrome sits within a wider need for integration of genetic testing into other cancer pathways and secondary care more generally. Thus, Lynch syndrome pathways should be congruent with the wider framework for local delivery of genomics.

### 2.2 Testing pathway analysis

Comprehensive pathway analysis was conducted in collaboration with the National Disease Registration Service (NDRS), which holds diagnostic and clinicopathological data for all cancer patients in England—over 500,000 new cases annually—including somatic and constitutional genetic testing outcomes for 44,000 CRC and EC cases.^20^ To support data‐driven improvement, NDRS developed a national dashboard allowing each NHS provider to access its own testing rates and identify local barriers to Lynch syndrome (LS) testing across CRC and EC pathways.

In 2023, a national audit was conducted involving all CRC and EC multidisciplinary teams (MDTs) in England to assess progress and identify ongoing development needs. While not covering all individual patients, the audit required each MDT to submit data on a minimum of 30 consecutive cancer diagnoses—to ensure unbiased data capture—and was coordinated locally by designated LS champions. This audit, based on a model previously shown to improve pathway performance,^21^ evaluated compliance with key standards: achieving >90% MMR testing in new cancer diagnoses and ensuring appropriate delivery of germline genetic testing in eligible patients. In two regions (North Thames and South East of England GMSAs), an additional audit was undertaken to facilitate a subgroup analysis of the time to from cancer diagnosis to time of genetic testing was assessed within and without the mainstreamed model.

Responsibility for audit accuracy was devolved to local teams, however regional project leads held audit review meetings to review the data, clarify anomalies, investigate pathway deviations, and agree outcomes and pathway improvements, and report comprehensive audit results to the national team.

Validation of overall effectiveness is available through quarterly releases of data showing the number of Lynch syndrome diagnoses made for each Cancer Alliance region. This is benchmarked to the number of expected Lynch syndrome diagnoses based on CRC and EC prevalence.

Ultimate validation of local unit performance will be provided the NDRS Lynch syndrome dashboard which is updated annually from the Cancer Outcomes and Statistics Dataset (COSD), but with a 2–4‐year delay for data validation and correlation.

Baseline data collected in 2021, including a national survey of LS champions, had revealed substantial geographical variation and a discrepancy between perceived and actual testing activity.^11^, ^18^ The most frequently reported barriers included unclear funding pathways and the absence of systematic approaches to testing. To measure progress, an end‐of‐project survey was conducted in early 2025.

### 2.3 Patient Ascertainment for the English National LS registry (ENLSR)

The LS Transformation Project also supported parallel national initiatives, including the establishment of an English National LS Registry (ENLSR) and the development of a dedicated LS Bowel Cancer Screening Programme (LS‐BCSP). Mainstream genetic testing is supported through multidisciplinary regional expert networks, with stakeholders which include regional genetics services.^22^

To populate the ENLSR, a retrospective data collection exercise was undertaken to identify all individuals historically diagnosed with LS across England. Each regional genetics service, along with the LS service at St Mark's Hospital—key diagnostic providers prior to project initiation—was asked to compile a list of historically diagnosed LS patients. A minimum dataset was developed, including essential identifiers (first name, surname, date of birth, NHS number, gender, and postcode), date of LS diagnosis, and LS gene. Additional desirable data fields included colonoscopy history, specific genetic variant and classification, full address, and ethnicity. This minimum dataset was designed to be sufficient to facilitate timely access to key interventions, including enrolment in the LS‐BCSP.

Data collection was performed by each of the 17 regional genetics services in England, plus St Mark's (National Bowel) Hospital, followed by validation and de‐duplication by the registry team. Regional genetics services were asked to mine local patient and laboratory information systems. Search terms were expansive to include older diagnostic terms such as HNPCC.

Genetics services and St Mark's Hospital were chosen, due to key favourable attributes:

- They provide complete National coverage for clinical care, regardless of whether the genetic test was performed in an NHS laboratory.
- They have been maintaining records for in excess of 35 years, predating digital systems, and incorporating the full historical period of diagnostic genetic testing for Lynch syndrome.
- They maintain family records, facilitating prospective ascertainment though pre‐symptomatic testing.

To support ongoing patient identification, NDRS developed an online portal for the submission of prospective LS diagnoses. All participating diagnostic services were asked to submit the same minimum dataset for newly diagnosed patients through this portal. In parallel, existing regional LS registries were reviewed to identify and address any data gaps. Diagnoses obtained via private laboratories were submitted retrospectively if known to Genetic services, and prospectively via the LS portal, for example, if patients move from abroad they are referred by their primary care doctor to a genetics service who will then enter their details. Data were securely shared with NDRS under the legal framework of Section 254 of the Health and Social Care Act 2012. This infrastructure provided the foundation for a robust, centralised LS registry that supports population‐level surveillance, targeted screening, and future service planning.

### 2.4 Statistical analysis

Analysis of local audit data was undertaken using descriptive analyses, and national analysis in coordination with NDRS. Chi‐squared tests were used to analyse binary variables within the survey.

## 3 RESULTS

The National LS Transformation Project included creation of a strong network of Lynch stakeholders and provided a critical forum for engagement, shared learning, discussion of existing issues and collaborative solution design. We initiated the establishment of local, regional and a national network to support local champions of whom 276 LS champions were appointed in CRC and EC teams (>95% coverage nationally), within 97% coverage of 143 CRC MDTs and 94% of 119 gynae‐oncology MDTs nationally.

In total, 658 online national training courses were completed by MDT members, with 125 modules partially completed (Table S2). The largest professional group completing module option 1 training were nurses for both options 1 (focused on pathway delivery, 50%) and option 2 (mainstreaming, 57%). In addition 207 cancer MDT members completed the pathology modules (67% of these were pathologists).

### 3.1 Performance of the diagnostic pathway

Comprehensive data along the LS testing pathway have been made available to NHS providers via the NDRS Dashboard. However, this dataset mainly reflects performance from 2022, in line with standard national reporting timelines for cancer registration and the typical two‐year lag between cancer diagnosis and the availability of germline genetic results. As such, it does not fully capture the impact of recent programme changes. To address this, audit data were used to assess programme outcomes in a more timely manner. Between 2022 and 2023, 99.2% (263 out of 265) of cancer MDTs in England completed this audit of a total of 7890 patients (i.e., 30 from each MDT), providing a near‐complete and contemporaneous picture of testing activity and pathway improvements.

#### 3.1.1 Tumour testing

**FIGURE 1:**
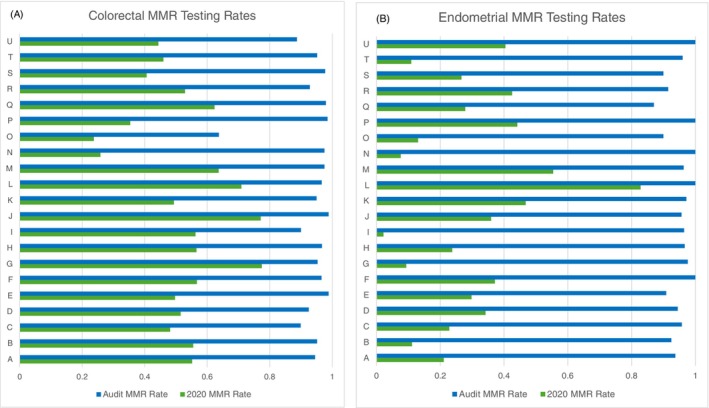
MMR testing rates for CRC (A) and EC (B) for each of 21 cancer alliances in England (anonymised, data available on request), comparing gold standard baseline data from 2021 and audit data from 2024.

Tumour MMR testing rates were compared between gold‐standard baseline data from NDRS from 2020, and ‘contemporary’ audit data of 30 consecutively diagnosed patients from each cancer MDT from 2022 to 2023 (Figure 1). Testing rates along the LS pathway significantly improved for both CRC and EC. Nationally, MMR testing rates increased from 43% to 94% for CRC and from 19% to 95% for EC. These improvements were observed consistently across all GMSA regions in England, with every region achieving MMR testing rates above 91% (range: 92–100%). For the minority of teams with MMR testing rates below 90%, re‐audits were arranged by the NHS Cancer Programme to support further improvement—specifically, 16 out of 143 CRC MDTs and 12 out of 119 EC MDTs in England.

#### 3.1.2 Constitutional (Germline) genetic testing

New diagnoses of people with Lynch syndrome (LS) in England have consistently increased each year since 2020. To support this, new mainstreaming services were established within MDTs and supported through regional expert networks—enabling genetic testing to be offered directly by oncology or surgical teams without the need for referral to a regional clinical genetics service. This approach integrates constitutional (germline) testing into routine cancer care, improving accessibility and reducing delays in diagnosis. By 2024, mainstreamed pathways were in place in 46% of CRC and 41% of gynaecological oncology teams across England, representing a major shift toward localised delivery of genomic medicine within multidisciplinary cancer services.

In sub‐group analysis of 109 consecutive mainstream cases from the North Thames GMSA region, the time from index somatic testing to constitutional testing was reduced to 21 days in mainstreamed patients, versus a minimum 180 days for those referred to regional clinical genetic services. Evidence from South East of England GMSA demonstrate a time to diagnosis reducing to 150–200 days, with further improvements anticipated as mainstreaming further embeds within pathways.

**FIGURE 2:**
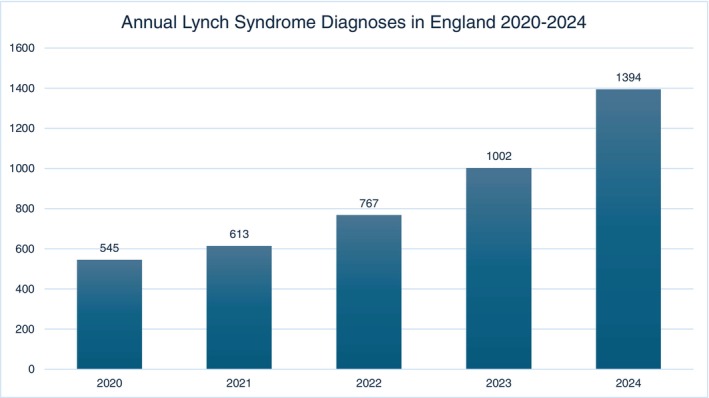
LS diagnoses by year 2020–2024 (data from NDRS).

NDRS developed an application programming interface (API) portal through which prospective diagnoses could be ascertained for a the ENLSR. In this way real time information on new diagnoses could be measured against historical published data collated by NDRS and ascertained thorough regional genomic laboratory hub services. In 2020 a total of 535 patients were diagnosed with LS in England, in 2024 this figure was 1394, an increase of 4% new diagnoses annually over the project lifetime (Figure 2).

Through this project, 9030 individuals previously diagnosed with LS have been identified and included in the ENLSR.^13^ Of these, 8471 are eligible for the nationally coordinated LS Bowel Cancer Screening Programme (LS‐BCSP), a world's first nationally coordinated risk‐stratified screening programme which was launched in 2023. As new LS diagnoses are made, they are entered into the ENLSR via a secure national portal, and data is then shared on a daily basis between the ENLSR in NDRS and the National Bowel Cancer Screening Programme‘s IT system (BCSS: Bowel Cancer Screening System), ensuring timely inclusion and facilitating access to targeted surveillance and preventive interventions.

### 3.2 Expert networks for LS

Multidisciplinary regional expert networks for Lynch syndrome (LS) were established across all seven GMSA regions in England. These networks were created to promote equity of access for individuals with LS, support the delivery and sustainability of mainstreamed testing pathways, and coordinate lifelong care for affected individuals.^18^, ^23^

Each network developed standard operating procedures and identified key stakeholders, including LS champions and relevant specialist services. Dedicated websites were created for each region, providing information on access to specialist LS clinics, regional multidisciplinary meetings, and opportunities for participation in LS‐related research studies.

To ensure accountability and consistency, the national oversight group defined a set of performance metrics to benchmark LS network activity. These included: the number of cancer MDTs per region offering mainstreamed germline testing, the level of training and implementation support available within each network, and the extent of multiprofessional engagement in LS service delivery.

### 3.3 End of project LS Champions survey

**FIGURE 3:**
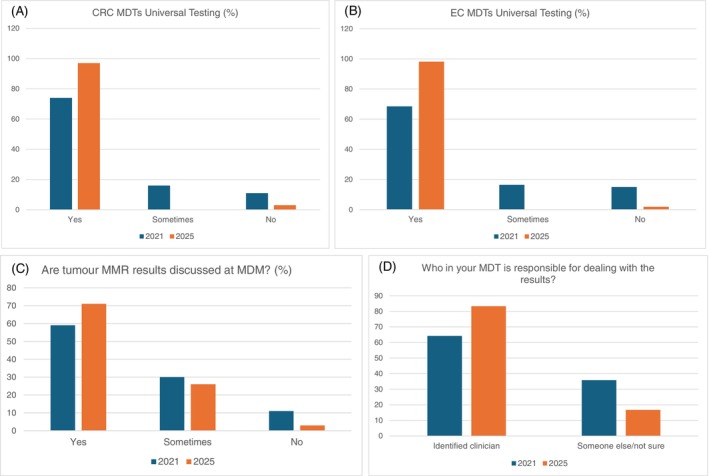
Outcome of survey of Lynch syndrome champions from 2021 and 2025. (a) Do you perform universal testing of all new colorectal cancer cases for LS at your Trust/site (with immunohistochemistry and/or MSI testing)? (b) Do you perform universal testing of all new endometrial cancer cases for LS at your Trust/site (with immunohistochemistry)? (c) Are all tumour MMR (mismatch repair) results discussed at MDM (MDT Meeting)? (d) Who in your MDT is responsible for dealing with the results?

In total 87 LS champions responded to the end of project survey between January to February 2025 (45 CRC, 32 Gynae‐Oncology), including 34 surgeons, 18 nurses, 4 oncologists, 5 pathologists and 16 other clinicians. Of these, 97% reported that universal tumour testing was provided by their MDT to CRC patients, and 98% EC patients, with IHC being the index test used in 74%, MSI in 20%, and the others reporting a blended approach. The results of the baseline ‘project initiation’ survey in 2021 were compared to the ‘end of project’ survey undertaken in 2025 to identify changes to practice (Figure 3).

Tumour MMR testing rates were reported to be significantly higher in 2025, for both CRC and EC, results were more likely to be discussed and documented at MDT meetings, with clearer responsibility for the specific MDT clinician identified who would arrange the next step in the testing pathway (83.3% in 2025 vs. 64.2% in 2021; *p* = <0.001).

When asked about changes in the delivery of germline genetic testing for LS, 88% of respondents reported an increase in testing, 10% noted no change, and <2% reported a decrease. In relation to how genetic testing pathways are managed locally, 44 respondents (57%) indicated that their teams were delivering in‐house mainstreamed genetic testing, while 15 teams (19%) reported they were in the process of transitioning to a mainstreamed model. The remaining teams continued to refer patients to regional clinical genetics services. Among those not offering mainstreamed testing, the most commonly cited barrier was related to clinical service commissioning arrangements, reported by 31% of respondents.

## 4 DISCUSSION

**FIGURE 4:**
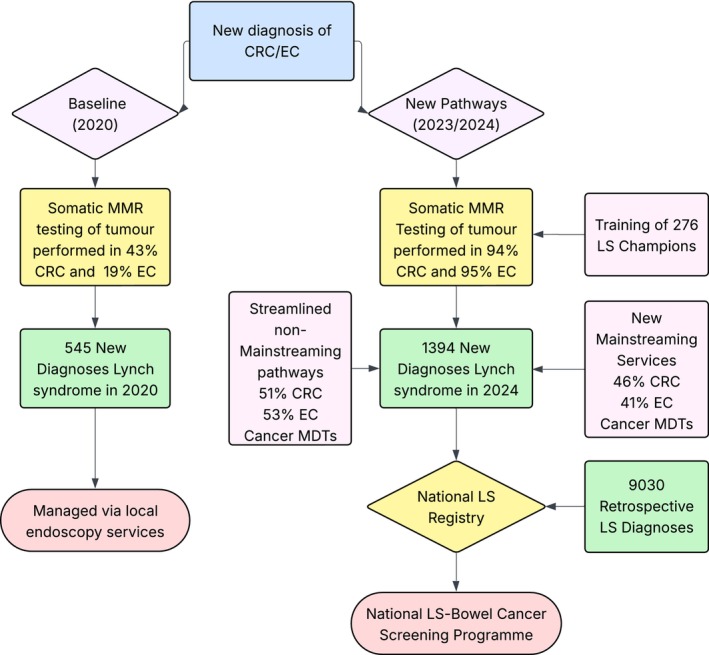
Summary of outcomes from the National Lynch Syndrome Transformation Project. CRC (Colorectal Cancer); EC (Endometrial Cancer); LS (Lynch syndrome); MDT (Multidisciplinary team).

The National LS Transformation Project aimed to establish robust, locally delivered testing pathways for LS, ensuring that responsibility for testing was clearly embedded within MDTs and supported by regional and national expertise. Prior to the project, testing rates among eligible patients were low and inconsistent across regions—an issue mirrored in other international health systems.^24^ Through this initiative, we achieved systematic improvement by embedding LS champions within each multidisciplinary team, delivering tailored training programmes, auditing local testing pathways, and introducing mainstreamed genetic testing models. This resulted in a marked increase in tumour MMR testing rates—from 43% to 94% for CRC and from 19% to 95% for EC—as well as reduced time to germline testing and diagnosis. Consistent and significant pathway improvements consistent were reported in end of project survey of LS champions. In addition a 255% increase in new LS diagnoses annually have been implemented in England, surpassing targets and expectations of this project (Figure 4).

Over 9000 individuals previously diagnosed with LS have now been captured within the English National Lynch Syndrome Registry (ENLSR), which combines both retrospectively diagnosed cases and prospective ascertainment of new diagnoses.^13^ Thus far in previously published study, we have demonstrated association of baseline testing with deprivation, ethnicity and other factors, as well as geographical disparities. We will continue work with NDRS and publish future outcomes from the ENLSR.^11^, ^12^, ^13^ The ENLSR underpins delivery of the first nationally coordinated, quality‐assured colonoscopy programme for LS (LS‐BCSP), enabling systematic risk‐reduction on a scale unprecedented worldwide. The success of this initiative has provided a model for broader application: in 2025, it directly informed the creation of the National Inherited Cancer Predisposition Register (NICPR), a comprehensive database encompassing carriers of approximately 120 cancer predisposition genes across England.^13^, ^25^

These gains were achieved through a coordinated approach where local MDTs took ownership of delivery, underpinned by a strong regional infrastructure and national oversight. The current multi‐step testing pathway can result in drop‐off at each stage. By training the workforce and embedding clear local responsibility through LS champions, the project has laid the groundwork for more efficient implementation of future models—such as single‐step paired somatic–germline testing.

A recent retrospective analysis across multiple UK centres showed significant under‐diagnosis: while mismatch repair (MMR) tumour testing was widely adopted, there was considerable attrition along the testing pathway, with fewer than half of eligible patients proceeding to germline testing. This highlights a clear argument for mainstreaming to prevent drop‐off and ensure timely Lynch syndrome diagnosis.^1^

The cost‐ and clinical‐effectiveness of genetic diagnosis of LS in cancer patients has been demonstrated in the UK population in NICE guidelines DG27 and DG42 which included a comprehensive economic evaluation, as well as through linked health technology assessments.^16^, ^17^, ^26^, ^27^ Without complete testing pathways, the cost‐effectiveness of tumour‐first screening strategies is compromised. A UK‐based economic evaluation demonstrated that reflex MMR immunohistochemistry and MLH1 methylation testing in endometrial cancer was only cost‐effective (~£14,200 per QALY gained) when followed by germline confirmation.^28^

A key finding from our work is that the median timescale from cancer diagnosis to genetic confirmation of Lynch syndrome is just under one year in England. The main bottleneck in this pathway is the referral to regional genetics services where capacity is limited.^29^, ^30^ However, when patients are tested locally under mainstreaming pathways, this overall timeline is reduced by over 150 days. The outcome of genetic testing may directly inform oncological management, with implications for survival and the potential to save lives—an essential objective for the NHS. For example, given approximately 20% of people with CRC will not survive 1 year post diagnosis, access to genetic testing is an imperative for eligible patients and their families.

Mainstreaming by gynaecology teams has recently been recommended in recent NICE ovarian cancer NICE guidelines.^31^ Clinical genetics services rely on referral of eligible patients for ascertainment, and cannot identify patients who are not formally referred by MDTs or other clinicians. Conversely MDTs will have regular interaction with the own patients, and have an existing trusted relationship, supported by cancer nurse specialist who have advanced communication skills training.

Patients referred externally to regional genetics services must often navigate new clinical teams and unfamiliar settings, which introduces barriers such as communication gaps and logistical delays, particularly when additional visits or blood draws are required. In contrast, when genetic testing is initiated by the cancer team, any required blood samples can be taken alongside routine investigations, streamlining care and improving patient experience. A systematic review by Bokkers et al. further supports the feasibility of mainstreaming, showing that when non‐genetic clinicians are appropriately supported, mainstream testing is both practical and maintains quality standards.^32^

Our national LS Transformation Project validates and extends these insights: by embedding LS champions, delivering targeted training, auditing pathway adherence, and enabling mainstreamed somatic–germline testing, we have overcome system bottlenecks and achieved sustained improvements in testing uptake and timeliness across England.

Patients who are referred externally to genetics services will have to negotiate new clinical teams, clinical sites and may have to overcome difficulties in communication. If a blood test is required special arrangements must be made for a patient to attend an unfamiliar environment, conversely if a blood test is required for genetic testing under a MDT, the patient will have this test alongside existing blood testing as part of their cancer management at diagnosis, or during treatment.

A key strength of our approach was enabling local and regional teams to identify their own barriers and implement tailored solutions suited to their geography and infrastructure. This flexibility allowed broad and rapid changes to occur across the country. Learning was shared across organisational boundaries, promoting collaboration and spread of effective practices. Nationally developed training resources, dashboards, and registries have laid the groundwork for long‐term monitoring and measurement of delivery. Additionally, partnerships with charities have significantly raised public awareness of Lynch syndrome. These organisations have played a critical role in improving understanding of hereditary cancer risk, supporting educational efforts, and helping to promote early detection and timely treatment—ultimately improving patient outcomes and saving lives.

It is important to acknowledge the limitations of this approach. Our baseline evaluation relied on retrospective comprehensive data of all CRC and EC diagnosed in England 2020, with a standard reporting timeline of 2 years. Albeit this was supported by contemporaneous audit data of consecutive patients, the prospective dataset was limited to 30 consecutive patients per cancer MDT, potentially limiting generalisability or introducing reporting bias. In some cases, the small sample sizes or desire to demonstrate improvement may have introduced positive reporting bias or limited generalisability. As such, while our findings are encouraging, they should be interpreted with caution and validated through ongoing prospective monitoring and evaluation. Nevertheless because consecutive cases were audited, selection of ‘successfully tested’ cases was avoided. In addition survey outcomes may represent subjective views of respondents. However, those who completed the end of project survey in 2025 were blinded to responses from 2021 therefore the survey results may be said to represent a perception of significant improvement which is consistent with relatively objective audit data outcomes, and higher numbers of patients diagnosed following germline testing available from the new national diagnostic portal created by NDRS.

It is also important to note that using ENSLR data as a proxy for new Lynch syndrome diagnoses has limitations. For retrospective data submitted prior to the launch of the API portal in January 2023, dates of diagnosis were variably recorded across services which may affect the completeness and temporal accuracy of case ascertainment. For prospective submissions, this risk has been reduced by making date of diagnosis a mandatory field as part of the data submission process.

To sustain progress, greater engagement with primary care is needed to support early recognition, long‐term monitoring, and continuity of care. Robust financial planning and clear incentives are also essential. Reliance on local audit is not sustainable; improvements in national data quality are required. While cascade testing remains a specialist genomics responsibility, lifelong care demands national registry oversight and regional multidisciplinary expertise. The NHS is well placed to deliver this through its standardised framework. However, divergence is emerging across the UK: in England, LS patients now benefit from a national, quality‐assured colonoscopy programme and structured support for aspirin uptake, but such care is not yet consistently available in the devolved nations.^33^

The English National Lynch Syndrome (LS) Transformation Project has embedded genomics into cancer MDTs across England, establishing mainstreaming teams, expanding access to diagnostic testing, and implementing NICE guidance (DG27 and DG42). These changes have delivered universal LS testing and reduced unwarranted variation in care. To sustain progress, mainstreamed LS testing must be nationally funded, supported by appropriate incentives, workforce training across care settings, and Regional Expert Networks to manage complex cases.

Ultimately, if the population at large is to benefit from advances in genomic medicine, genetics services cannot do this alone,^34^ therefore we have instituted a mainstreaming model in England. The transformation achieved in England shows what is possible—but it remains fragile, and unequal access across the UK is already creating disparities in care. Without sustained national funding and commitment, the benefits of this project will be limited to where investment continues, leaving patients elsewhere behind.

## AUTHOR CONTRIBUTIONS

**Kevin J. Monahan:** Conceptualization; investigation; funding acquisition; writing – original draft; methodology; formal analysis; project administration; data curation; supervision; resources. **Paul Fleming:** Resources; writing – original draft. **Neil A. J. Ryan:** Writing – original draft; conceptualization; methodology; validation; visualization; resources. **Laura Monje‐Garcia:** Conceptualization; investigation; writing – original draft; methodology; resources; project administration. **Ruth Armstrong:** Resources; conceptualization; investigation; funding acquisition; writing – original draft; project administration. **David N. Church:** Writing – original draft; funding acquisition; investigation; conceptualization; project administration. **Jackie Cook:** Writing – original draft; funding acquisition; investigation; conceptualization; project administration. **Fiona Lalloo:** Conceptualization; investigation; funding acquisition; writing – original draft; project administration. **Sally Lane:** Conceptualization; resources. **Frank D. McDermott:** Conceptualization; investigation; writing – original draft; funding acquisition; resources; project administration. **Tracie Miles:** Conceptualization; resources. **Corinne Mallinson:** Data curation. **Steven A. Hardy:** Conceptualization; resources; data curation; writing – original draft. **Simone Gelinas:** Conceptualization; investigation; funding acquisition; project administration. **Francesca Faravelli:** Conceptualization; investigation; funding acquisition; project administration. **Frances Elmslie:** Project administration; conceptualization; investigation; funding acquisition. **Adam C. Shaw:** Conceptualization; investigation; funding acquisition; writing – original draft; writing – review and editing; project administration; resources.

## FUNDING INFORMATION

This project was funded by the Genomics Unit and Cancer Departments at NHS England. Kevin J. Monahan receives funding from the charity 40tude Curing Bowel Cancer for research in Lynch syndrome. David N. Church is funded by a Cancer Research UK (CRUK) Advanced Clinician Scientist Fellowship (C26642/A27963) Veracyte, Moderna, GSK.

## CONFLICT OF INTEREST STATEMENT

David N. Church speaker fees GSK. David N. Church Other: spouse Amgen employee. *All unrelated to current manuscript. All other authors declare no conflict of interest.

## Supporting information


**TABLE S1.** Training module participation by cancer MDT members.
**TABLE S2.** Baseline key performance indictors (KPIs) for year 1 of the National Lynch Syndrome Transformation Programme.

## Data Availability

The datasets generated and/or analysed during the current study are available from the corresponding author on reasonable request.
