# Supplementary material for: Outcomes from the English National Lynch Syndrome transformation project
Source: Int J Cancer. 2026 Jan 11;158(9):2369–79. doi: 10.1002/ijc.70330 (PMC12963710; doi:10.1002/ijc.70330)
Supplement: Supplementary file 1 — TABLE S1. Training module participation by cancer MDT members. TABLE S2. Baseline key performance indictors (KPIs) for year 1 of the National Lynch Syndrome Transformation Programme. [file IJC-158-2369-s001.pdf]

Outcomes from the English National Lynch Syndrome Transformation Project

Supplementary Tables

Authors:

Kevin J Monahan, Paul Fleming, Neil AJ Ryan, Laura Monje-Garcia, Ruth Armstrong, David N Church, Jackie Cook, Fiona Lalloo, Sally Lane, Frank D McDermott, Tracie Miles, Corinne Mallinson, Steven A Hardy, Simone Gelinas Francesca Faravelli, Frances Elmslie, Adam C Shaw

Contents

Supplementary table 1: Training module participation by cancer MDT members.

Supplementary table 2: Baseline key performance indictors (KPIs) for year 1 of the National Lynch Syndrome Transformation Programme

Suppl Table 1: Training module participation by cancer MDT members. Option 1 refers to training in a streamlining process for effective identification and referral of patients eligible for germline diagnostic testing, Option 2 refers to Mainstreaming training where germline testing is performed 'in house'

Module set	Completed	`Partial
Training option 1	278	54
Training option 2	205	39
Training option 3 Pathology CRC Training	97	27
Training option 4 Pathology EC	78	5

Suppl Table 2: Baseline key performance indictors (KPIs) for year 1 of the National Lynch Syndrome Transformation Programme

- A 50% increase on testing across each step of the testing pathway (MMR tumour testing, methylation or BRAF testing, and constitutional genetic testing) compared to baseline level measured by NDRS
- Appointment of a Lynch syndrome champion in each CRC and EC MDT
- Completion of a qualitative baseline survey of perceptions of testing level, barriers and solutions by each champion
- Development of standardisation of reporting for pathology
- Development of a rapid registration cancer dataset by NDRS (designed to provide a monitoring tool to continuously assess testing performance)
- Identification of all diagnosed Lynch syndrome patients by each GMSA (within their own geography)
- Development of electronic informatics and testing applications through collaboration.
- Development of online training modules and national training workshops for members of the CRC or EC MDTs, primary care and pathologists.
- At each monthly national oversight meeting a set of actions were agreed for GMSAs and CAs respectively.
